# Adenosine usage during AF ablation in Europe and selected long-term findings from the ESC-EHRA EORP Atrial Fibrillation Ablation Long-Term registry

**DOI:** 10.1007/s10840-020-00744-8

**Published:** 2020-04-30

**Authors:** Frank van Rosmalen, Tammo Delhaas, Nikolaos Dagres, Elena Arbelo, Carina Blomström-Lundqvist, Harry J. G. M. Crijns, Antoine Da Costa, Mariusz Pytkowski, Nikita Sharikov, Cécile Laroche, Luigi Tavazzi, Joseph Brugada, Laurent Pison, on behalf of the ESC-EHRA Atrial Fibrillation Ablation Long-Term Registry investigators

**Affiliations:** 1 Department of Cardiology, Maastricht University Medical Center, PO Box 5800, 6202 AZ Maastricht, The Netherlands; 2 Department of Biomedical Engineering, Maastricht University, Maastricht, The Netherlands; 3 Cardiovascular Research Institute Maastricht (CARIM), Maastricht, The Netherlands; 4 Department of Electrophysiology, Heart Center Leipzig, Leipzig, Germany; 5 Department of Cardiology, Cardiovascular Institute, Hospital Clinic de Barcelona, Universitat de Barcelona, Institut d’Investigació August Pi i Sunyer (IDIBAPS), Barcelona, Spain; 6 Centro de Investigación Biomédica en Red de Enfermedades Cardiovasculares (CIBERCV), Madrid, Spain; 7 Department of Medical Science and Cardiology, Uppsala University, Uppsala, Sweden; 8 C.H.U. de Saint Etienne, Hôpital Nord, Saint-Priest-en-Jarez, France; 9 Heart Rhythm Division of the 2nd Department of Coronary Artery Disease, Institute of Cardiology, Warszawa, Poland; 10 Department of Surgical Treatment of Complex Rhythm Disturbances and Electro-cardio-stimulation, District Clinical Hospital, Khanty-Mansiysk, Autonomous Okrug-Yugra Russia; 11 EURObservational Research Programme (EORP), Scientific Division, European Society of Cardiology, Sophia Antipolis, France; 12 GVM Care and Research, E.S., Maria Cecilia Hospital, Cotignola, Italy; 13 Cardiovascular Institute, Hospital Clínic Pediatric Arrhythmia Unit, Hospital Sant Joan de Déu University of Barcelona, Barcelona, Spain; 14 Department of Cardiology, Ziekenhuis Oost Limburg, Genk, Belgium

**Keywords:** Adenosine, Pulmonary vein isolation, Rhythm outcome, Safety, Follow-up

## Abstract

**Background:**

Adenosine can be used to reveal dormant pulmonary vein (PV) conduction after PV isolation (PVI). This study presents a subanalysis of real-world 1-year follow-up data from the ESC-EHRA EORP Atrial Fibrillation (AF) Ablation Long-Term registry to analyze the usage of adenosine during PVI treatment in terms of rhythm outcome and safety.

**Methods:**

The registry consists of 104 participating centers in 27 countries within the European Society of Cardiology. The registry data was split into an adenosine group (AG) and no-adenosine group (NAG). Procedure characteristics and patient outcome were compared.

**Results:**

Adenosine was administered in 10.8% of the 3591 PVI patients included in the registry. Spain, the Netherlands, and Italy included the majority of adenosine cases (48.8%). Adenosine was applied more often in combination with open irrigation radiofrequency (RF) energy (74.7%) and less often in combination with nonirrigated RF energy (1.6%). After 1 year, a higher percentage of the AG was free from AF compared with the NAG (68.9% vs 59.1%, *p* < 0.001). Adenosine was associated with better rhythm outcome in RF ablation procedures, but not in cryo-ablation procedures (freedom from AF: RF: AG: 70.9%, NAG: 58.1%, *p* < 0.001, cryo: AG: 63.9%, NAG: 63.8%, *p* = 0.991).

**Conclusions:**

The use of adenosine was associated with a better rhythm outcome after 1 year follow-up and seems more useful in patients treated with RF energy compared with patients treated with cryo energy. Given the improved rhythm outcome at 1-year follow-up, it seems reasonable to encourage the use of adenosine during RF AF ablation.

## Introduction

Ablation of the pulmonary veins (PVs) or atrium ideally results in irreversible cell damage and subsequent cell death, but can also temporarily cause reversible cell damage leading to a lower resting potential. Consequently, these nondestroyed cells are more difficult to activate, causing a transient electrical block [1]. Once the resting potential of the nondestroyed cells is restored, the “dormant conduction” revives. This revival might lead to reconnection of one or more PVs, which is the leading cause of arrhythmia recurrence after PVI [2].

It was shown that adenosine can be used to reveal dormant conduction of the PVs after PV isolation by increasing the resting potential of PV cells [1]. Publications on the usefulness and long-term outcome of adenosine use after PVI have come from relatively small clinical trials, observational studies, or randomized controlled trials with conflicting results [3]. The current guidelines for the management of atrial fibrillation consider adenosine testing to identify the need for additional ablation to be controversial [4].

This study presents a subanalysis of the large ESC EORP Atrial Fibrillation Ablation (AFA) Long-Term registry consisting of real-world 1-year follow-up data to analyze the effects of adenosine use during PVI treatment in terms of rhythm outcome and safety.

## Methods

All data were collected from the Atrial Fibrillation Ablation Long-Term registry, a prospective, multinational study (not a randomized clinical trial—RCT) conducted by the EURObservational Research Programme (EORP). The ESC EORP AFA Long-Term registry was previously described elsewhere [5]. In short, it is a prospective, multicenter observational study of consecutive patients undergoing an ablation procedure for atrial fibrillation (AF) at one of the 104 participating centers in 27 countries within the European Society of Cardiology. All patients included in the registry were enrolled between April 2012 and April 2015 with a maximum of 50 consecutive patients per center. Out of all patients enrolled in the registry, patients with an unclassified type of AF, patients who did not undergo an ablation procedure, or patients treated with both radiofrequency (RF) and cryo energy in the same procedure were excluded to be able to perform the analyses presented in this paper. All patients signed an informed consent before data collection and were followed up for 1 year according to the local clinical practice. The protocol was approved by the national and/or local Institutional Review Boards, according to existing regulations in each country. The registry management, central data quality control, and the statistical analysis were performed by the EORP Center of the ESC, which coordinated also a random local auditing of 22% participating centers across 14 countries. The registry data was split into one group of patients where adenosine was used to check for dormant conduction (adenosine group: AG) and another group of patients where adenosine was not used (no adenosine group: NAG). Since this was an observational registry in which the patients were treated according to the local protocol, the application of adenosine during the ablation procedure as well as the timing of testing after ablation, dosage of adenosine, or additional use of isoproterenol were all left at the discretion of the operator. Since adenosine administration was based on the local protocol, it is assumed that adenosine was used with the intention to treat and therefore for detecting reconnections and ablation of these reconnections when present. It is assumed that additional ablation is performed in PVs only since adenosine is able to reveal dormant conduction in PV cells but not in LA cells [1]. For the 1 year clinical follow-up data, the registry was first split into a cryoballoon group and a radiofrequency energy group and then into an AG and NAG in order to assess the possible influence of different ablation techniques (point-by-point RF vs cryoballoon) on rhythm outcome.

The 1-year follow-up data of patients enrolled in the in-hospital phase was used for the analyses. Continuous variables were reported as mean ± standard deviation (SD) or as median with interquartile range (IQR). Group comparisons were made using a nonparametric test (Kruskal–Wallis test). Categorical variables were reported as percentages. Group comparisons were made using a chi-square test or Fisher’s exact test (if any expected cell count was < 5). Plots of the Kaplan-Meier curves for arrhythmia-free survival according to type of AF categories were generated. The survival distributions were compared using the log-rank test. A two-sided *p* value < 0.05 was considered statistically significant. All analyses were performed using SAS Statistical Software version 9.4 (SAS Institute, Inc., Cary, NC, USA).

## Results

In the total number of 3591 patients that underwent AF ablation, adenosine was administered in 367 (10.2%) patients to unmask dormant conduction. A total of 152 patients (4.2%) were excluded because both RF and cryo energy was used in the same procedure, resulting in 3439 patients included for the analysis, of which 360 were treated with adenosine. A large difference between countries was noted. Most adenosine cases were included by centers in Spain, followed by the Netherlands and Italy (respectively 27.5%, 11.2%, and 10.1% of total included adenosine cases), whereas centers in Bulgaria, Denmark, Israel, Kazakhstan, Latvia, Romania, and Slovenia did not include any adenosine cases (see Table 1).

**Table 1:** Adenosine use in Europe

	Total cases	Adenosine	
		Total (% of total within country)	% of total within Europe
Spain	526	101 (19.2)	27.5
Netherlands	242	41 (16.9)	11.2
Italy	314	37 (11.8)	10.1
Austria	41	27 (65.9)	7.4
United Kingdom	48	22 (45.8)	6.0
Russian Federation	467	21 (4.5)	5.7
Belgium	84	20 (23.8)	5.5
Sweden	108	14 (13.0)	3.8
Ukraine	87	13 (14.9)	3.5
Finland	170	13 (7.7)	3.5
Czech Republic	79	11 (13.9)	3.0
Greece	139	9 (6.5)	2.5
Ireland	28	8 (28.6)	2.2
Portugal	44	7 (15.9)	1.9
Poland	338	7 (2.1)	1.9
Hungary	60	5 (8.3)	1.4
Egypt	78	5 (6.4)	1.4
France	152	3 (2.0)	0.8
Germany	204	2 (1.0)	0.5
Belarus	20	1 (5.0)	0.3
Bulgaria	107	0 (0.0)	0.0
Denmark	17	0 (0.0)	0.0
Israel	53	0 (0.0)	0.0
Kazakhstan	30	0 (0.0)	0.0
Latvia	35	0 (0.0)	0.0
Romania	61	0 (0.0)	0.0
Slovenia	66	0 (0.0)	0.0

The AG and NAG consisted of 96 (26.7%) and 654 (21.2%) redo patients, respectively. After 1 year, 3179 patients (96.8%) were still in follow-up (10.1% AG, 89.9% NAG). Loss to follow-up was 12.8% for the AG and 11.3% for the NAG.

### Practice of adenosine use

Although there was a significant difference in longstanding persistent AF between the groups (*p* = 0.004), the ratio of paroxysmal, persistent, and long-standing persistent was similar, with the longstanding AF group accounting for a minority of cases in both groups (see Table 2). The AG consisted of 71.7% paroxysmal, 26.4% persistent, and 1.9% long-standing persistent AF patients, whereas the NAG consisted of 66.9% paroxysmal, 27.6% persistent, and 5.5% long-standing persistent AF patients (see Table 2).

**Table 2:** Baseline characteristics

	Cryo or RF energy			*p*	Cryo energy			*p*	RF energy			*p*
	Total	Adenosine	No adenosine		Total	Adenosine	No adenosine		RF	Adenosine	No adenosine	
Gender (female)	1096/3439 (31.9%)	92/360 (25.6%)	1004/3079 (32.6%)	0.007 (S)	185/543 (34.1%)	17/74 (23.0%)	168/469 (35.8%)	0.030(S)	911/2896 (31.5%)	75/286 (26.2%)	836/2610 (32.0%)	0.045(S)
Age ≥ 65 years (mean, SD)	910/3438 (26.5%)	86/360 (23.9%)	824/3078 (26.8%)	0.241 (NS)	154/543 (28.4%)	22/74 (29.7%)	132/469 (28.1%)	0.779(NS)	57.8 (10.3)	56.1 (10.6)	58.0 (10.2)	0.004 (S)
Body Mass Index (kg/m2) (mean, SD)	28.4 (4.5)	27.9 (4.3)	28.5 (4.5)	0.101 (NS)	28.1 (3.9)	27.6 (3.8)	28.1 (3.9)	0.535 (NS)	28.5 (4.6)	28.0 (4.4)	28.5 (4.6)	0.143 (NS)
Left atrial diameter (mm) (mean, SD)	42.6 (6.6)	42.1 (5.4)	42.7 (6.7)	0.310 (NS)	41.0 (5.9)	41.1 (4.5)	41.0 (6.0)	0.633 (NS)	59.9 (8.4)	59.9 (7.7)	59.9 (8.5)	0.080 (NS)
Type of AF												
Paroxysmal	2318/3439 (67.4%)	258/360 (71.7%)	2060/3079 (66.9%)	0.068(NS)	454/543 (83.6%)	66/74 (89.2%)	388/469 (82.7%)	0.163(NS)	1864/2896 (64.4%)	192/286 (67.1%)	1672/2610 (64.1%)	0.303(NS)
Persistent	946/3439 (27.5%)	95/360 (26.4%)	851/3079 (27.6%)	0.615(NS)	79/543 (14.5%)	8/74 (10.8%)	71/469 (15.1%)	0.326(NS)	867/2896 (29.9%)	87/286 (30.4%)	780/2610 (29.9%)	0.851(NS)
Longstanding persistent	175/3439 (5.1%)	7/360 (1.9%)	168/3079 (5.5%)	0.004(S)	10/543 (1.8%)	0/74 (0.0%)	10/469 (2.1%)	0.372(NS)	165/2896 (5.7%)	7/286 (2.4%)	158/2610 (6.1%)	0.013(S)

*AF* atrial fibrillation, *NS* non significant, *S* significant, *SD* standard deviation

The AG had a lower number of ablations of fractionated electrogram sites in both the left (AG: 5.1%, NAG: 8.9%, *p* = 0.015) and right (AG:0.8%, NAG: 3.6%, *p* = 0.007) atrium, as well as fewer left atrial roof lines (AG:7.9%, NAG: 14.0%, *p* = 0.002) and mitral isthmus lines (AG:3.1%, NAG: 7.0%, *p* = 0.005) (see Table 3).

**Table 3:** Type of procedure and ablation strategy

	Cryo or RF energy			*p*	Cryo energy			*p*	RF energy			*p*
	Total	Adenosine	No adenosine		Total	Adenosine	No adenosine		RF	Adenosine	No adenosine	
Type of procedure												
First procedure	2685/3439 (78.1%)	263/360 (73.1%)	2422/3079 (78.7%)	0.015(S)	490/543 (90.2%)	70/74 (94.6%)	420/469 (89.6%)	0.174(NS)	2195/2896 (75.8%)	193/286 (67.5%)	2002/2610 (76.7%)	< 0.001(S)
Redo due to Atrial Fibrillation	660/3439 (19.2%)	94/360 (26.1%)	566/3079 (18.4%)	< 0.001(S)	49/543 (9.0%)	4/74 (5.4%)	45/469 (9.6%)	0.242(NS)	611/2896 (21.1%)	90/286 (31.5%)	521/2610 (20.0%)	< 0.001(S)
Redo due to Left Atrial Flutter/Left Atrial Tachycardia	90/3439 (2.6%)	2/360 (0.6%)	88/3079 (2.9%)	0.010(S)	4/543 (0.7%)	0/74 (0.0%)	4/469 (0.9%)	1.000(NS)	86/2896 (3.0%)	2/286 (0.7%)	84/2610 (3.2%)	0.017(S)
Hybrid	4/3439 (0.1%)	1/360 (0.3%)	3/3079 (0.1%)	0.358(NS)	0/543 (0.0%)	0/74 (0.0%)	0/469 (0.0%)	–	4/2896 (0.1%)	1/286 (0.3%)	3/2610 (0.1%)	0.340(NS)
Ablation strategy												
Fractionated electrogram sites in the left atrium	278/3263 (8.5%)	18/353 (5.1%)	260/2910 (8.9%)	0.015(S)	8/533 (1.5%)	0/74 (0.0%)	8/459 (1.7%)	0.607(NS)	270/2730 (9.9%)	18/279 (6.5%)	252/2451 (10.3%)	0.042(S)
Fractionated electrogram sites in right atrium	107/3263 (3.3%)	3/353 (0.8%)	104/2910 (3.6%)	0.007(S)	4/533 (0.8%)	0/74 (0.0%)	4/459 (0.9%)	1.000(NS)	103/2730 (3.8%)	3/279 (1.1%)	100/2451 (4.1%)	0.013(S)
Left atrial linear lesion												
Roof line	436/3264 (13.4%)	28/353 (7.9%)	408/2911 (14.0%)	0.002(S)	0/533 (0.0%)	0/74 (0.0%)	0/459 (0.0%)	NA	436/2731 (16.0%)	28/279 (10.0%)	408/2452 (16.6%)	0.004(S)
Mitral isthmus line	216/3264 (6.6%)	11/353 (3.1%)	205/2911 (7.0%)	0.005(S)	2/533 (0.4%)	0/74 (0.0%)	2/459 (0.4%)	1.000(NS)	214/2731 (7.8%)	11/279 (3.9%)	203/2452 (8.3%)	0.011(S)
Posterior line	137/3199 (4.3%)	16/342 (4.7%)	121/2857 (4.2%)	0.702(NS)	0/513 (0.0%)	0/74 (0.0%)	0/439 (0.0%)	NA	137/2686 (5.1%)	16/268 (6.0%)	121/2418 (5.0%)	0.495(NS)
Other left atrial linear lesion	99/3264 (3.0%)	6/353 (1.7%)	93/2911 (3.2%)	0.122(NS)	0/533 (0.0%)	0/74 (0.0%)	0/459 (0.0%)	NA	99/2731 (3.6%)	6/279 (2.2%)	93/2452 (3.8%)	0.164(NS)
Achievement of entrance block												
LSPV	3004/3104 (96.8%)	328/334 (98.2%)	2676/2770 (96.6%)	0.118(NS)	510/528 (96.6%)	69/73 (94.5%)	441/455 (96.9%)	0.294(NS)	2494/2576 (96.8%)	259/261 (99.2%)	2235/2315 (96.5%)	0.019(S)
LIPV	2939/3030 (97.0%)	323/330 (97.9%)	2616/2700 (96.9%)	0.320(NS)	498/516 (96.5%)	66/71 (93.0%)	432/445 (97.1%)	0.087(NS)	2441/2514 (97.1%)	257/259 (99.2%)	2184/2255 (96.9%)	0.031(S)
RSPV	2984/3094 (96.4%)	327/334 (97.9%)	2657/2760 (96.3%)	0.127(NS)	494/519 (95.2%)	67/72 (93.1%)	427/447 (95.5%)	0.371(NS)	2490/2575 (96.7%)	260/262 (99.2%)	2230/2313 (96.4%)	0.015(S)
RIPV	2953/3083 (95.8%)	323/330 (97.9%)	2630/2753 (95.5%)	0.045(S)	480/507 (94.7%)	67/71 (94.4%)	413/436 (94.7%)	0.781(NS)	2473/2576 (96.0%)	256/259 (98.8%)	2217/2317 (95.7%)	0.014(S)
Adenosine												
Use of adenosine at end of procedure	353/3264 (10.8%)	353/353 (100.0%)	0/2911 (0.0%)	< 0.001(S)	74/533 (13.9%)	74/74 (100.0%)	0/459 (0.0%)	< 0.001(S)	279/2731 (10.2%)	279/279 (100.0%)	0/2452 (0.0%)	< 0.001(S)
Adenosine-induced reconnection of PV	61/353 (17.3%)	61/353 (17.3%)	0/0 (0.0%)	–	15/74 (20.3%)	15/74 (20.3%)	0/0 (0.0%)	–	46/279 (16.5%)	46/279 (16.5%)	0/0 (0.0%)	–
Catheter used												
Nonirrigated RF	44/3439 (1.3%)	1/360 (0.3%)	43/3079 (1.4%)	0.082(NS)	0/543 (0.0%)	0/74 (0.0%)	0/469 (0.0%)	NA	44/2896 (1.5%)	1/286 (0.3%)	43/2610 (1.6%)	0.121(NS)
RF with closed irrigation	125/3439 (3.6%)	12/360 (3.3%)	113/3079 (3.7%)	0.747(NS)	0/543 (0.0%)	0/74 (0.0%)	0/469 (0.0%)	NA	125/2896 (4.3%)	12/286 (4.2%)	113/2610 (4.3%)	0.916(NS)
RF with open irrigation	2730/3439 (79.4%)	273/360 (75.8%)	2457/3079 (79.8%)	0.078(NS)	0/543 (0.0%)	0/74 (0.0%)	0/469 (0.0%)	NA	2730/2896 (94.3%)	273/286 (95.5%)	2457/2610 (94.1%)	0.363(NS)
Cryo	543/3439 (15.8%)	74/360 (20.6%)	469/3079 (15.2%)	0.009(S)	543/543 (100.0%)	74/74 (100.0%)	469/469 (100.0%)	NA	0/2896 (0.0%)	0/286 (0.0%)	0/2610 (0.0%)	NA
Duty-cycled RF energy	9/3439 (0.3%)	0/360 (0.0%)	9/3079 (0.3%)	0.610(NS)	0/543 (0.0%)	0/74 (0.0%)	0/469 (0.0%)	NA	9/2896 (0.3%)	0/286 (0.0%)	9/2610 (0.3%)	1.000(NS)
High intensity focused ultrasound	8/3439 (0.2%)	0/360 (0.0%)	8/3079 (0.3%)	1.000(NS)	0/543 (0.0%)	0/74 (0.0%)	0/469 (0.0%)	NA	8/2896 (0.3%)	0/286 (0.0%)	8/2610 (0.3%)	1.000(NS)
Laser balloon	0/3439 (0.0%)	0/360 (0.0%)	0/3079 (0.0%)	NA	0/543 (0.0%)	0/74 (0.0%)	0/469 (0.0%)	NA	0/2896 (0.0%)	0/286 (0.0%)	0/2610 (0.0%)	NA
Other	3/3 (100.0%)	0/0 (0.0%)	3/3 (100.0%)	NA	–	–	–	–	3/3 (100.0%)	0/0 (0.0%)	3/3 (100.0%)	NA
Procedure												
Procedure duration (min) (mean, SD)	165.9 (65.8)	161.2 (55.3)	166.5 (66.9)	0.566 (NS)	138.3 (49.3)	162.6 (60.3)	134.4 (46.3)	< 0.001 (S)	170.9 (67.1)	160.8 (54.2)	172.0 (68.4)	0.042 (S)

*LIPV* left inferior pulmonary vein, *LSPV* left superior pulmonary vein, *NA* not applicable, *NS* non significant, *PV* pulmonary vein, *RF* radio frequency, *RIPV* right inferior pulmonary vein, *RSPV* right superior pulmonary vein, *S* significant, *SD* standard deviation

Whereas the overall use of adenosine was 10.8%, adenosine was applied more often in combination with procedures where open irrigation RF energy is used (79.4%, *p*), while it is less often used in combination with nonirrigated RF energy (0.3%) or duty-cycled RF energy (0%) (see Table 3). Cryo energy was relatively overrepresented in the AG (AG: 20.6%, NAG: 15.2%, *p* = 0.009).

### Procedural effects of adenosine

The use of adenosine did not cause a significantly higher total procedure time (measured from the moment of catheter placement until the moment of catheter removal). There was no significant difference in achievement of only entrance block between the AG and NAG, but all PVs showed significantly more entrance and exit block in the AG when compared with the NAG (see Table 3).

Administration of adenosine revealed reconnection of PVs in 17.3% of adenosine cases.

### Effects of adenosine usage on patient outcome

#### Adverse events

Procedural adverse events potentially related to adenosine use were rare. The adenosine group showed a higher occurrence of pericarditis (AG: 1.7%, NAG: 0.7%, *p* = 0.048) and pulmonary vein stenosis (AG: 0.6%, NAG: 0.0%, *p* = 0.031, defined as a reduction of the diameter of a PV or PV branch of more than 50%). There was no significant difference for other general, cardiovascular, gastrointestinal, neurological, peripheral/vascular, or pulmonary adverse events (Table 5 in the Appendix).

Adverse events at 1 year are listed in Table 6 in the Appendix. One infrequently occurring difference in adverse events at 1 year follow-up was found to be phrenic nerve damage (AG: 2/304, NAG: 0/2669).

#### One year follow-up

Follow-up at 12 months after the index procedure showed a significant difference in rhythm outcome between the AG and NAG; a higher percentage of the AG was free from AF compared with the NAG (AG: 69.2%, NAG: 58.9%, *p* < 0.001). Observing a 3-month-blanking period, the AG showed a lower percentage of arrhythmia recurrence during the first year after the index procedure compared with the NAG (AG: 20.8%, NAG: 26.3%, *p* = 0.038) (see Table 4).

**Table 4:** One-year clinical follow-up

	Cryo or RF energy			*p*	Cryo energy			*p*	RF energy			*p*
	Total	Adenosine	No adenosine		Total	Adenosine	No adenosine		Total	Adenosine	No adenosine	
Classification of AF 1 year after the procedure												
Permanent AF	61/3010 (2.0%)	5/308 (1.6%)	56/2702 (2.1%)	0.596(NS)	7/493 (1.4%)	0/71 (0.0%)	7/422 (1.7%)	0.601(NS)†	54/2517 (2.1%)	5/237 (2.1%)	49/2280 (2.1%)	0.968(NS)
Persistent AF	295/3010 (9.8%)	25/308 (8.1%)	270/2702 (10.0%)	0.294(NS)	25/493 (5.1%)	2/71 (2.8%)	23/422 (5.5%)	0.558(NS)†	270/2517 (10.7%)	23/237 (9.7%)	247/2280 (10.8%)	0.593(NS)
Paroxysmal AF	825/3010 (27.4%)	62/308 (20.1%)	763/2702 (28.2%)	0.003(S)	145/493 (29.4%)	24/71 (33.8%)	121/422 (28.7%)	0.380(NS)	680/2517 (27.0%)	38/237 (16.0%)	642/2280 (28.2%)	< 0.001(S)
Longstanding persistent AF	25/3010 (0.8%)	3/308 (1.0%)	22/2702 (0.8%)	0.737(NS)†	4/493 (0.8%)	0/71 (0.0%)	4/422 (0.9%)	1.000(NS)†	21/2517 (0.8%)	3/237 (1.3%)	18/2280 (0.8%)	0.441(NS)†
No AF	1804/3010 (59.9%)	213/308 (69.2%)	1591/2702 (58.9%)	< 0.001(S)	312/493 (63.3%)	45/71 (63.4%)	267/422 (63.3%)	0.986(NS)	1492/2517 (59.3%)	168/237 (70.9%)	1324/2280 (58.1%)	< 0.001(S)
Recurrence (after blanking period)	762/2958 (25.8%)	64/307 (20.8%)	698/2651 (26.3%)	0.038(S)	123/478 (25.7%)	18/68 (26.5%)	105/410 (25.6%)	0.880(NS)	639/2480 (25.8%)	46/239 (19.2%)	593/2241 (26.5%)	0.015(S)
Method of documentation												
ECG	804/3029 (26.5%)	66/311 (21.2%)	738/2718 (27.2%)	0.025(S)	120/492 (24.4%)	16/71 (22.5%)	104/421 (24.7%)	0.694(NS)	684/2537 (27.0%)	50/240 (20.8%)	634/2297 (27.6%)	0.025(S)
Hospital discharge record	189/3025 (6.2%)	13/311 (4.2%)	176/2714 (6.5%)	0.112(NS)	32/490 (6.5%)	2/71 (2.8%)	30/419 (7.2%)	0.294(NS)†	157/2535 (6.2%)	11/240 (4.6%)	146/2295 (6.4%)	0.277(NS)
Transtelephonic monitoring	23/3025 (0.8%)	0/311 (0.0%)	23/2714 (0.8%)	0.161(NS)†	6/490 (1.2%)	0/71 (0.0%)	6/419 (1.4%)	0.600(NS)†	17/2535 (0.7%)	0/240 (0.0%)	17/2295 (0.7%)	0.397(NS)†
Written diagnosis	167/3025 (5.5%)	13/311 (4.2%)	154/2714 (5.7%)	0.274(NS)	18/490 (3.7%)	2/71 (2.8%)	16/419 (3.8%)	1.000(NS)†	149/2535 (5.9%)	11/240 (4.6%)	138/2295 (6.0%)	0.370(NS)
Holter	380/3026 (12.6%)	33/311 (10.6%)	347/2715 (12.8%)	0.274(NS)	67/491 (13.6%)	12/71 (16.9%)	55/420 (13.1%)	0.388(NS)	313/2535 (12.3%)	21/240 (8.8%)	292/2295 (12.7%)	0.075(NS)
Implanted monitoring system	57/3025 (1.9%)	5/311 (1.6%)	52/2714 (1.9%)	0.705(NS)	15/490 (3.1%)	0/71 (0.0%)	15/419 (3.6%)	0.144(NS)†	42/2535 (1.7%)	5/240 (2.1%)	37/2295 (1.6%)	0.591(NS)†
Management of recurrence												
Cardioversion	459/1020 (45.0%)	31/85 (36.5%)	428/935 (45.8%)	0.099(NS)	63/158 (39.9%)	4/22 (18.2%)	59/136 (43.4%)	0.025(S)	396/862 (45.9%)	27/63 (42.9%)	369/799 (46.2%)	0.610(NS)
Redo ablation	223/996 (22.4%)	17/82 (20.7%)	206/914 (22.5%)	0.707(NS)	41/154 (26.6%)	3/21 (14.3%)	38/133 (28.6%)	0.169(NS)	182/842 (21.6%)	14/61 (23.0%)	168/781 (21.5%)	0.792(NS)
Cardiology visit	2507/3031 (82.7%)	256/312 (82.1%)	2251/2719 (82.8%)	0.745(NS)	418/493 (84.8%)	48/71 (67.6%)	370/422 (87.7%)	< 0.001(S)	2089/2538 (82.3%)	208/241 (86.3%)	1881/2297 (81.9%)	0.087(NS)
Emergency room visit	472/3030 (15.6%)	45/312 (14.4%)	427/2718 (15.7%)	0.553(NS)	82/493 (16.6%)	10/71 (14.1%)	72/422 (17.1%)	0.533(NS)	390/2537 (15.4%)	35/241 (14.5%)	355/2296 (15.5%)	0.701(NS)
Hospitalization due to arrhythmia in blanking period	206/420 (49.0%)	12/25 (48.0%)	194/395 (49.1%)	0.914(NS)	29/67 (43.3%)	2/4 (50.0%)	27/63 (42.9%)	1.000(NS)†	177/353 (50.1%)	10/21 (47.6%)	167/332 (50.3%)	0.812(NS)
Hospitalization due to arrhythmia outside blanking period	320/420 (76.2%)	14/25 (56.0%)	306/395 (77.5%)	0.015(S)	52/68 (76.5%)	2/4 (50.0%)	50/64 (78.1%)	0.233(NS)†	268/352 (76.1%)	12/21 (57.1%)	256/331 (77.3%)	0.035(S)
Hospitalization due to other cardiovascular event	96/2969 (3.2%)	9/309 (2.9%)	87/2660 (3.3%)	0.736(NS)	11/492 (2.2%)	3/71 (4.2%)	8/421 (1.9%)	0.202(NS)†	85/2477 (3.4%)	6/238 (2.5%)	79/2239 (3.5%)	0.417(NS)
Hospitalization due to noncardiovascular event	166/2970 (5.6%)	20/309 (6.5%)	146/2661 (5.5%)	0.475(NS)	31/492 (6.3%)	2/71 (2.8%)	29/421 (6.9%)	0.289(NS)†	135/2478 (5.4%)	18/238 (7.6%)	117/2240 (5.2%)	0.130(NS)
Follow-up duration (months, mean (SD))	13.0 (3.6)	12.3 (2.4)	13.1 (3.7)	0.004(S)	12.8 (2.8)	12.7 (2.5)	12.8 (2.9)	< 0.001(S)†	13.0 (3.7)	12.2 (2.3)	13.1 (3.8)	0.179(NS)
Number of repeat procedures												
1	257/2958 (8.7%)	22/303 (7.3%)	235/2655 (8.9%)	0.662(NS)†	46/479 (9.6%)	4/68 (5.9%)	42/411 (10.2%)	0.482(NS)†	211/2479 (8.5%)	18/235 (7.7%)	193/2244 (8.6%)	0.930(NS)†
2	21/2958 (0.7%)	1/303 (0.3%)	20/2655 (0.8%)		4/479 (0.8%)	0/68 (0.0%)	4/411 (1.0%)		17/2479 (0.7%)	1/235 (0.4%)	16/2244 (0.7%)	
3	3/2958 (0.1%)	0/303 (0.0%)	3/2655 (0.1%)		–	–	–		3/2479 (0.1%)	0/235 (0.0%)	3/2244 (0.1%)	

The 1 year follow-up rhythm outcome was split into a cryoballoon group (493 patients) and a RF energy (nonirrigated radiofrequency, radiofrequency with closed irrigation, or radiofrequency with open irrigation) group (2517 patients). Patients where both cryo and RF energy was used within the same procedure were excluded from the analysis (152 patients). While there was no significant difference in rhythm outcome or arrhythmia recurrence between the AG and NAG for patients treated with cryo energy, the RF energy group showed less paroxysmal AF patients in the AG group (AG: 16.0%, NAG: 28.2%, *p* < 0.001), more patients without AF (AG: 70.9%, NAG: 58.1%, *p* < 0.001), and a lower number of arrhythmia recurrences (AG: 19.2%, NAG: 26.5%, *p* < 0.015) after 1 year follow-up (see Table 4).

There was no significant difference in the number of redo procedures between the AG and NAG (*p* = 0.662) or within the cryo and RF (*p* = 0.482, *p* = 0.930) groups during the first year after the procedure (see Table 4).

## Discussion

This real-world registry shows adenosine to be used in only a small number (10.2%) of AF ablation procedures within the 104 participating centers of the ESC-EHRA Atrial Fibrillation Ablation Long-Term registry of the European Society of Cardiology and that there are large regional differences in adenosine use within Europe. The registry shows significant differences in freedom from arrhythmia after 1 year and in the number of arrhythmia-related hospitalizations, both in favor of the AG. The very low overall incidence of complications precludes further explanation regarding the cause of these adverse events. Adenosine seems to be more useful in RF patients compared with patients treated with cryo energy.

The low number of adenosine cases might be explained by the adherence to guidelines. Adenosine use was not covered in the guidelines available at the time of inclusion. At the time of writing, the guidelines describe adenosine as “may be considered” [2] and “controversial.” [4]

Multiple studies indicated no significant difference in terms of arrhythmia recurrence between using a cryoballoon or RF catheter. Our results, however, showed that adenosine use was associated with a difference in rhythm outcome between cryoballoon and RF: patients treated with RF energy seem to benefit from adenosine use, whereas patients treated with cryo energy did not. Application of point-by-point RF energy may be technically more challenging compared with the delivery of cryo energy with the use of a cryoballoon. This might lead to more gaps in the ablation line, which is associated with lower long-term ablation success [6]. Adenosine reveals these gaps, which might explain the difference in outcome between the AG and NAG in the RF group. This outcome is in contrast to the UNDER-ATP study by Kobori et al. [7] In this study, 2113 AF patients were randomized to RF energy PVI with vs without adenosine. Rhythm outcome after 1 year follow-up was not statistically different. A possible explanation for this discrepancy can be found in the baseline characteristics of our study. Whereas Kobori et al. report no significant differences in baseline characteristics between the two groups for paroxysmal, persistent, and long-standing AF, our AG had a small but significantly higher percentage of paroxysmal AF patients and a small but significantly lower percentage of long-standing persistent AF patients when compared with the NAG. The differences in baseline characteristics can be explained by the different study designs. Whereas Kobori et al. performed a prospective randomized trial, our study contains data from a retrospective registry and is more focused on adenosine usage, in comparison with outcome.

The ADVICE trial is a multicenter, randomized superiority trial consisting of 534 patients who underwent PVI with adenosine treatment at the end of the procedure [8]. Patients showing dormant conduction were randomly assigned to additional adenosine-guided ablation or to no further ablation, whereas patients not showing dormant conduction were followed in a separate registry. After 1 year, there was a significant difference between the groups regarding freedom from any atrial tachyarrhythmia after a single procedure, both with and without the use of anti-arrhythmic drugs. The dormant conduction group with additional ablation outperformed the nondormant conduction group, which, in turn, outperformed the dormant conduction group without further ablation. Because the patients in our study who received adenosine after PVI either had no dormant conduction or the reconnected PV was reisolated, we can compare our results with the “dormant conduction with additional ablation group” combined with the “no dormant conduction registry group” of the ADVICE trial. The ADVICE trial reports that after 1 year, 63.4% of patients where adenosine was used (the dormant conduction with additional ablation group combined with the no dormant conduction registry group) are free from any atrial tachyarrhythmia after a single procedure. This is comparable with our finding of 68.9% freedom of AF for the AG.

The differences between the UNDER-ATP study, the ADVICE trial, and our registry indicate that further research should be undertaken to investigate the effect of adenosine on arrhythmia recurrence after PVI. This should also answer the question whether adenosine should be used in combination with cryoballoon, RF, or both since the registry shows that the use of adenosine is not associated with improved rhythm outcome in combination with cryoballoon procedures but is currently used more often with cryoballoon procedures than RF procedures.

The UNDER-ATP study and ADVICE trial are responsible for the majority of cases included in two recent systematic reviews and meta-analysis [3, 9]. The meta-analysis of Wang et al. included 22 comparative studies and randomized controlled trials. Although there was significant heterogeneity between investigated studies, the authors conclude that the presence of PV reconnections identified with adenosine (and not ablated) result in poorer rates of freedom from AF, and that administration of adenosine post PVI improves rates of freedom from AF by identifying areas for further ablation. Wang et al. show a comparable outcome with our results (freedom from AF: AG: 67%, NAG: 63% (Wang et al.), versus AG: 68.9%, NAG: 59.1% (this article)). Afzal et al. present a meta-analysis over all RCTs investigating adenosine and PVI. The study included 5 RCTs available, all using RF catheters. The conclusion is that routine use of adenosine to guide PVI does neither improve arrhythmia-free survival nor need for repeat ablation. As noted by the authors of both meta-analyses, their outcome depended on the methodology of the observed studies, and there is a large variation in methods. Patient selection, PVI procedure, adenosine dosage, waiting time after adenosine administration, follow-up duration, and follow-up method are (among others) variables that ideally should be controlled for. Our results add to the growing body of data by showing that adenosine testing can improve outcome in specific cases in a large, multicenter real-world cohort.

A significantly higher occurrence of phrenic nerve damage at 1-year follow-up was observed in the AG, although the absolute numbers are low. Since phrenic nerve damage is reported as the most frequent adverse event for cryoballoon ablations [10, 11], the fact that cryo energy was used more often in the AG could have caused this difference in adverse events outcome.

If adenosine reveals dormant conduction, extra ablation is needed to close the line isolating the PVs. These extra ablations increase the energy delivered to the PVs, which, in turn, increases the chance of pericarditis and PV stenosis. This can be a reason for the increase in pericarditis and PV stenosis occurrence in the AG.

The fact that cryo patients do not seem to benefit from the use of adenosine might partially be a procedural reason. Most cryo ablations are PVI-only ablations and do not use a catheter able to target regions outside of the PV, nor make linear lesions. However, we compare these with RF ablations where adenosine is used, and in this group, only a minority (61 out of 353) received LA linear lesions*.*

Force sensing catheters and ablation index technology were not widely used during the inclusion period of this registry. The use of these techniques might decrease the need for adenosine testing as research indicates that the quality of point-by-point RF lesions increases significantly by using force sensing catheters and ablation index technology [12, 13].

## Limitations

The main limitations of this registry have already been described previously [5]. A prospective design requiring consecutive inclusion of patients was used to minimize the risk of selection and reporting bias. The EURObservational Research Programme Department of the ESC performed extensive validation and invited external auditors to assure quality and reliability of the data, as mentioned in the “methods” section. The interpretation of recurrence data may be limited by inhomogeneous and partially insufficient arrhythmia monitoring. Follow-up *at* 1 year was performed in 52.8% by clinical visit, 44.2% by telephone contact, and 3.0% by contact with the general practitioner, and although 82.7% of patients had a clinical visit *within* the first year, ideally all patients would have had 1 year follow-up by clinical visit and Holter monitoring to discover possible asymptomatic recurrence.

The ESC-EHRA EORP Atrial Fibrillation Ablation Long-Term registry was not designed to answer specific questions regarding adenosine dosage, timing, or strategy since the criteria for adenosine use, dosage, timing, and strategy were all left at the discretion of the operator. This precludes detailed conclusions about specific adenosine strategies.

Another limitation of the registry is that it contains no explicit information whether additional ablation was performed when PV reconnections where found. Since adenosine administration was based on the local protocol, it is assumed that adenosine was used with the intention to treat and therefore for detecting reconnections and ablation of these reconnections when present.

Because this registry presents observational data, the possibility of confounding factors that may influence the descriptive study results cannot be excluded. Because of the very low incidence of complications in this registry, it is not possible to provide evidence on possible correlations between complications, ablation techniques, and adenosine use. The main focus of the analysis is the use of adenosine within Europe. Since this is not a randomized controlled trial, small but significant differences in baseline characteristics between the AG and NAG might influence the results.

## Conclusion

In conclusion, this observational study shows that, though adenosine is used in only a small number of AF ablation cases, its use is associated with a better rhythm outcome in patients treated with RF energy. Given the very low incidence of complications associated with the use of adenosine, it seems reasonable to encourage the use of adenosine during RF energy AF ablation. These findings need to be confirmed in randomized controlled trials.

## Appendix 1. Committees and Investigators

Executive Committee

Nikolaos Dagres, Josep Brugada, Elena Arbelo, Luigi Tavazzi, Carina Blomström Lundqvist, Evgeny Pokushalov, Josef Kautzner, Aldo P. Maggioni.

Steering Committee (National Coordinators)

Clemens Steinwender, Alexandr Chasnoits, Georges Mairesse, Tosho Balabanski, Josef Kautzner, Sam Riahi, Mostafa Nawar, Mervat Abul El Maaty, Pekka Raatikainen, Frederic Anselme, Thorsten Lewalter, Turgut Brodherr, Michalis Efremidis, Laszlo Geller, Ben Glover, Roy Beinart, Michael Glikson, Fiorenzo Gaita, Roin Rekvava, Oskars Kalejs, Serge Trines, Zbigniew Kalarus, Mario Martins Oliveira, Pedro Adragao, Radu Ciudin, Evgeny Mikhaylov, Matjaz Sinkovec, Julian Perez Villacastin, Carina Blomström-Lundqvist, Oleg Sychov, Paul Roberts.

Investigators

**AUSTRIA** *Graz* D Daniel Scherr; Martin Manninger; Bernadette Mastnak; *Innsbruck* Otamr Pachinger; Florian Hintringer; Markus Stühlinger; *Linz* Clemens Steinwender; **BELGIUM** *Yvoir* Olivier Xhaet; **BULGARIA** *Sofia* Tchavdar Shalganov; Milko Stoyanov; Mihail Protich; *Sofia* Vassil Traykov; Daniel Marchov; Genadi Kaninski; **BELARUS** *Minsk* Alexandr Chasnoits; **CZECH REPUBLIC** *Prague* Robert Cihak; *Hradec Kralove* Ludek Haman; **GERMANY** *Frankfurt* Boris Schmidt; K.R. Julian Chun; Laura Perrotta; Stefano Bordignon; *Hamburg* Roland Tilz; *Hamburg* Stephan Willems; *Leipzig* Gerhard Hindricks; *München* Turgut Brodherr; Ilia S. Koutsouraki; Thorsten Lewalter; **DENMARK** *Aalborg* Sam Riahi; Bodil Ginnerup Sørensen; **EGYPT** *Cairo* Wagdi Galal; *Cairo* Amir AbdelWahab; *Cairo* S Sherif Mokhtar; **FINLAND** *Turku* Juha Lund; *Tampere* Pekka Raatikainen; **FRANCE** *Grenoble* Pascal Defaye; Peggy Jacon; Sandrine Venier; Florian Dugenet; *Saint Denis* Olivier Piot; Xavier Copie; Olivier Paziaud; Antoine Lepillier; *Saint Etienne* Antoine Da Costa; Cécile Romeyer-Bouchard; *Toulouse* Serge Boveda; Jean-Paul Albenque; Nicolas Combes; Stéphane Combes *Marseille* Ange Ferracci; André Pisapia; **GREECE** *Athens* Demosthenes Katritsis; *Athens* Konstantinos Letsas; Kostas Vlachos; Louiza Lioni; *Thessaloniki* Vassilios P. Vassilikos; **HUNGARY** *Budapest* Laszlo Geller, Nándor Szegedi; Gábor Széplaki; Tamás Tahin; *Debrecen* Zoltan Csanadi; Gabor Sandorfi; Alexandra Kiss; Edina Nagy-Balo; *Szeged* Laszlo Saghy; **IRELAND** *Dublin* Benedict M. Glover; Joseph Galvin; Edward Keelan; **ISRAEL** *Ramat* Roy Beinart; Michael Glikson; Eyal Nof; **ITALY** *Acquaviva delle Fonti* Massimo Grimaldi; Federico Quadrini; Antonio Di Monaco; Federica Troisi; *Castellanza* Massimo Tritto; Elvira Renzullo; Antonio Sanzo; Domenico Zagari; *Cotignola* Carlo Pappone; *Crema* Pietro Maria Giovanni Agricola; *Milano* Paolo Della Bella; *Napoli* Giuseppe Stabile; Assunta Iuliano; *Pisa* Maria Grazia Bongiorni; *Roma* Leonardo Calo; Ermenegildo de Ruvo; Luigi Sciarra; *Torino* Matteo Anselmino; Fiorenzo Gaita; Federico Ferraris; *Varese* Roberto De Ponti; Raffaella Marazzi; Lorenzo A. Doni; **KAZAKHSTAN** *Almaty* Roin Rekvava; Anna Kim; **LATVIA** *Riga* Oskars Kalejs; **NETHERLANDS** *Breda* Sander Molhoek; *Groningen* Isabelle Van Gelder; Michiel Rienstra; *Leiden* Serge Trines; Marieke G. Compier; *Maastricht* Laurent Pison; Harry J. Crijns; Kevin Vernooy; Justin Luermans; *Rotterdam* Luc Jordaens; Natasja de Groot; Tamas Szili-Torok; Rohit Bhagwandien; *Zwolle* Arif Elvan; Thomas Buist; Pim Gal; **POLAND** *Lodz* Andrzej Lubinski; *Gdansk* Tomasz Krolak; *Katowice* Seweryn Nowak, Katarzyna Mizia-Stec; Anna Maria Wnuk-Wojnar; *Krakow* Jacek Lelakowski; *Szczecin* Jaroslaw Kazmierczak; *Warszawa* Piotr Kulakowski; Jakub Baran; *Warszawa* Grzegorz Opolski; Marek Kiliszek; Piotr Lodziński; Sonia Borodzicz; Paweł Balsam; *Poznan* Krzysztof Blaszyk; *Warszawa* Mariusz Pytkowski; Rafal Kuteszko; Jan Ciszewski; *Wroclaw* Artur Fuglewicz; *Zabrze* Zbigniew Kalarus; Aleksandra Woźniak; Karolina Adamczyk; **PORTUGAL** *Carnaxide Lisboa* Pedro Adragao; *Lisboa* Pedro Cunha; **ROMANIA** *Iasi* Mihaela Grecu; Grigore Tinica; *Cluj-Napoca* Lucian Muresan; Radu Rosu; **RUSSIAN FEDERATION** *Kemerovo* Egor Khomenko; *Khanty-Mansiysk* Nikita Scharikov; *Krasnoyarsk* Dmitry Zamanov; *Krasnoyarsk* Evgenii Kropotkin; *Novosibirsk* Evgeny Pokushalov; Alexander Romanov; Sevda Bayramova; *Saint-Petersburg* Evgeny N. Mikhaylov; Dmitry S. Lebedev; Anna V. Patsouk; *Saint-Petersburg* Sergey Yashin; *Saint-Petersburg* Dmitry Kryzhanovskiy; *Saransk* Vyacheslav Bazayev; *Surgut* Denis Morgunov; Ilya Silin; *Tomsk* Sergey Popov; *Tyumen* Vadim Kuznetsov; **SPAIN** *Alicante* Ignacio Gil Ortega; Juan Gabriel Martinez Martinez; *Badajoz* Manuel Doblado Calatrava; *Barcelona* Roger Villuendas Sabate; *Barcelona* Lluis Mont Girbau; *Bilbao* Maria Fe Arcocha; Larraitz Gaztañaga; Estibaliz Zamarreño*; Granada* Miguel Álvarez; Rosa Macías; *Las Palmas de Gran Canaria* Federico Segura Villalobos; Juan Carlos Rodríguez Pérez; *Madrid* Nicasio Perez Castellano; Victoria Cañadas; Juan J Gonzalez Ferrer; David Filgueiras; *Madrid* Jose Manuel Rubio Campal; Pepa Sánchez-Borque; Juan Benezet-Mazuecos; *Madrid* Jorge Toquero Ramos; Fernandez Lozano; Victor Castro Urda; *Malaga* Alberto Barrera Cordero; Carmen Medina Palomo; Amalio Ruiz-Salas; Javier Alzueta; *Madrid* Rafael Peinado; David Filqueiras-Rama; Alfonso Gómez Gallanti; Daniel Garófalo; *Pamplona* Naiara Calvo; *Santander* Juan Jose Olalla Antolin; *Sevilla* Alonso Pedrote; Eduardo Arana-Rueda; Lorena García-Riesco; **SWEDEN** *Linköping* Anders Jönsson; *Lund* Pyotr Platonov; Fredrik Holmqvist; Ole Kongstad; Shiwen Yuan; *Umeå* Niklas Höglund; *Uppsala* Helena Malmborg; David Mörtsell; **SLOVENIA** *Ljubljana* Matjaz Sinkovec; Andrej Pernat; **UNITED KINGDOM** *Southampton* John Morgan; Paul Roberts; Elizabeth F. Greenwood; Lisa L. Fletcher; **UKRAINE** *Donetsk* Tetiana Kravchenko; *Kiev* Alexander Doronin; Maryna Meshkova; *Odessa* Iurii Karpenko; Alex Goryatchiy; Anna Abramova.

## Appendix 2

**Table 5:** Adverse events

	Cryo or RF energy			*p*	Cryo energy			*p*	RF energy			*p*
	Total	Adenosine	No adenosine		Total	Adenosine	No adenosine		RF	Adenosine	No adenosine	
Cardiovascular												
Cardiac arrest	3/3430 (0.1%)	0/360 (0.0%)	3/3070 (0.1%)	1.000(NS)†	0/543 (0.0%)	0/74 (0.0%)	0/469 (0.0%)	NA	3/2887 (0.1%)	0/286 (0.0%)	3/2601 (0.1%)	1.000(NS)†
Air embolism	8/3430 (0.2%)	1/360 (0.3%)	7/3070 (0.2%)	0.589(NS)†	3/543 (0.6%)	0/74 (0.0%)	3/469 (0.6%)	1.000(NS)†	5/2887 (0.2%)	1/286 (0.3%)	4/2601 (0.2%)	0.407(NS)†
Endocarditis	0/3430 (0.0%)	0/360 (0.0%)	0/3070 (0.0%)	NA	0/543 (0.0%)	0/74 (0.0%)	0/469 (0.0%)	NA	0/2887 (0.0%)	0/286 (0.0%)	0/2601 (0.0%)	NA
Myocardial infarction	0/3430 (0.0%)	0/360 (0.0%)	0/3070 (0.0%)	NA	0/543 (0.0%)	0/74 (0.0%)	0/469 (0.0%)	NA	0/2887 (0.0%)	0/286 (0.0%)	0/2601 (0.0%)	NA
Cardiac perforation	46/3430 (1.3%)	1/360 (0.3%)	45/3070 (1.5%)	0.084(NS)†	7/543 (1.3%)	0/74 (0.0%)	7/469 (1.5%)	0.601(NS)†	39/2887 (1.4%)	1/286 (0.3%)	38/2601 (1.5%)	0.174(NS)†
Bradycardia requiring pacemaker	5/3431 (0.1%)	0/360 (0.0%)	5/3071 (0.2%)	1.000(NS)†	0/543 (0.0%)	0/74 (0.0%)	0/469 (0.0%)	NA	5/2888 (0.2%)	0/286 (0.0%)	5/2602 (0.2%)	1.000(NS)†
Cardiac thromboembolic event	2/3430 (0.1%)	1/360 (0.3%)	1/3070 (0.0%)	0.199(NS)†	1/543 (0.2%)	0/74 (0.0%)	1/469 (0.2%)	1.000(NS)†	1/2887 (0.0%)	1/286 (0.3%)	0/2601 (0.0%)	0.099(NS)†
Heart valve damage	1/3430 (0.0%)	0/360 (0.0%)	1/3070 (0.0%)	1.000(NS)†	0/543 (0.0%)	0/74 (0.0%)	0/469 (0.0%)	NA	1/2887 (0.0%)	0/286 (0.0%)	1/2601 (0.0%)	1.000(NS)†
Pericarditis	26/3431 (0.8%)	6/360 (1.7%)	20/3071 (0.7%)	0.048(S)†	0/543 (0.0%)	0/74 (0.0%)	0/469 (0.0%)	NA	26/2888 (0.9%)	6/286 (2.1%)	20/2602 (0.8%)	0.038(S)†
Other	50/3434 (1.5%)	8/360 (2.2%)	42/3074 (1.4%)	0.200(NS)	3/543 (0.6%)	0/74 (0.0%)	3/469 (0.6%)	1.000(NS)†	47/2891 (1.6%)	8/286 (2.8%)	39/2605 (1.5%)	0.132(NS)†
General												
Allergic reaction	9/3435 (0.3%)	1/360 (0.3%)	8/3075 (0.3%)	1.000(NS)†	3/543 (0.6%)	0/74 (0.0%)	3/469 (0.6%)	1.000(NS)†	6/2892 (0.2%)	1/286 (0.3%)	5/2606 (0.2%)	0.465(NS)†
Sepsis	1/3436 (0.0%)	0/360 (0.0%)	1/3076 (0.0%)	1.000(NS)†	0/543 (0.0%)	0/74 (0.0%)	0/469 (0.0%)	NA	1/2893 (0.0%)	0/286 (0.0%)	1/2607 (0.0%)	1.000(NS)†
Gastrointestinal												
Esophageal ulceration	2/3437 (0.1%)	1/360 (0.3%)	1/3077 (0.0%)	0.199(NS)†	0/543 (0.0%)	0/74 (0.0%)	0/469 (0.0%)	NA	2/2894 (0.1%)	1/286 (0.3%)	1/2608 (0.0%)	0.188(NS)†
Esophageal fistula or perforation	1/3437 (0.0%)	0/360 (0.0%)	1/3077 (0.0%)	1.000(NS)†	0/543 (0.0%)	0/74 (0.0%)	0/469 (0.0%)	NA	1/2894 (0.0%)	0/286 (0.0%)	1/2608 (0.0%)	1.000(NS)†
Neurological												
Stroke	1/3437 (0.0%)	0/360 (0.0%)	1/3077 (0.0%)	1.000(NS)†	0/543 (0.0%)	0/74 (0.0%)	0/469 (0.0%)	NA	1/2894 (0.0%)	0/286 (0.0%)	1/2608 (0.0%)	1.000(NS)†
TIA	8/3436 (0.2%)	1/360 (0.3%)	7/3076 (0.2%)	0.588(NS)†	3/543 (0.6%)	0/74 (0.0%)	3/469 (0.6%)	1.000(NS)†	5/2893 (0.2%)	1/286 (0.3%)	4/2607 (0.2%)	0.406(NS)†
Phrenic nerve damage	13/3436 (0.4%)	4/360 (1.1%)	9/3076 (0.3%)	0.039(S)†	11/543 (2.0%)	4/74 (5.4%)	7/469 (1.5%)	0.050(S)†	2/2893 (0.1%)	0/286 (0.0%)	2/2607 (0.1%)	1.000(NS)†
Vagal nerve injury	0/3436 (0.0%)	0/360 (0.0%)	0/3076 (0.0%)	NA	0/543 (0.0%)	0/74 (0.0%)	0/469 (0.0%)	NA	0/2893 (0.0%)	0/286 (0.0%)	0/2607 (0.0%)	NA
Peripheral nerve injury	0/3436 (0.0%)	0/360 (0.0%)	0/3076 (0.0%)	NA	0/543 (0.0%)	0/74 (0.0%)	0/469 (0.0%)	NA	0/2893 (0.0%)	0/286 (0.0%)	0/2607 (0.0%)	NA
Peripheral/vascular												
AV fistula	14/3435 (0.4%)	2/360 (0.6%)	12/3075 (0.4%)	0.652(NS)†	1/542 (0.2%)	0/74 (0.0%)	1/468 (0.2%)	1.000(NS)†	13/2893 (0.4%)	2/286 (0.7%)	11/2607 (0.4%)	0.373(NS)†
DVT	0/3433 (0.0%)	0/360 (0.0%)	0/3073 (0.0%)	NA	0/542 (0.0%)	0/74 (0.0%)	0/468 (0.0%)	NA	0/2891 (0.0%)	0/286 (0.0%)	0/2605 (0.0%)	NA
Peripheral thromboembolic event	0/3433 (0.0%)	0/360 (0.0%)	0/3073 (0.0%)	NA	0/542 (0.0%)	0/74 (0.0%)	0/468 (0.0%)	NA	0/2891 (0.0%)	0/286 (0.0%)	0/2605 (0.0%)	NA
Pseudoaneurysm	16/3433 (0.5%)	1/360 (0.3%)	15/3073 (0.5%)	1.000(NS)†	3/542 (0.6%)	0/74 (0.0%)	3/468 (0.6%)	1.000(NS)†	13/2891 (0.4%)	1/286 (0.3%)	12/2605 (0.5%)	1.000(NS)†
Hematoma or bleeding requiring evacuation or transfusion	12/3434 (0.3%)	0/360 (0.0%)	12/3074 (0.4%)	0.627(NS)†	4/543 (0.7%)	0/74 (0.0%)	4/469 (0.9%)	1.000(NS)†	8/2891 (0.3%)	0/286 (0.0%)	8/2605 (0.3%)	1.000(NS)†
Bleeding requiring transfusion	4/3433 (0.1%)	2/360 (0.6%)	2/3073 (0.1%)	0.057(NS)†	1/542 (0.2%)	1/74 (1.4%)	0/468 (0.0%)	0.137(NS)†	3/2891 (0.1%)	1/286 (0.3%)	2/2605 (0.1%)	0.268(NS)†
Pulmonary												
Hemothorax	1/3437 (0.0%)	0/360 (0.0%)	1/3077 (0.0%)	1.000(NS)†	0/543 (0.0%)	0/74 (0.0%)	0/469 (0.0%)	NA	1/2894 (0.0%)	0/286 (0.0%)	1/2608 (0.0%)	1.000(NS)†
Pneumothorax	2/3437 (0.1%)	0/360 (0.0%)	2/3077 (0.1%)	1.000(NS)†	0/543 (0.0%)	0/74 (0.0%)	0/469 (0.0%)	NA	2/2894 (0.1%)	0/286 (0.0%)	2/2608 (0.1%)	1.000(NS)†
Pulmonary vein damage/dissection	0/3437 (0.0%)	0/360 (0.0%)	0/3077 (0.0%)	NA	0/543 (0.0%)	0/74 (0.0%)	0/469 (0.0%)	NA	0/2894 (0.0%)	0/286 (0.0%)	0/2608 (0.0%)	NA
Pulmonary vein stenosis	3/3437 (0.1%)	2/360 (0.6%)	1/3077 (0.0%)	0.031(S)†	0/543 (0.0%)	0/74 (0.0%)	0/469 (0.0%)	NA	3/2894 (0.1%)	2/286 (0.7%)	1/2608 (0.0%)	0.027(S)†
Pleural effusion	5/3437 (0.1%)	1/360 (0.3%)	4/3077 (0.1%)	0.425(NS)†	1/543 (0.2%)	0/74 (0.0%)	1/469 (0.2%)	1.000(NS)†	4/2894 (0.1%)	1/286 (0.3%)	3/2608 (0.1%)	0.341(NS)†
Pulmonary embolism	0/3437 (0.0%)	0/360 (0.0%)	0/3077 (0.0%)	NA	0/543 (0.0%)	0/74 (0.0%)	0/469 (0.0%)	NA	0/2894 (0.0%)	0/286 (0.0%)	0/2608 (0.0%)	NA
Pulmonary vein thrombosis	0/3437 (0.0%)	0/360 (0.0%)	0/3077 (0.0%)	NA	0/543 (0.0%)	0/74 (0.0%)	0/469 (0.0%)	NA	0/2894 (0.0%)	0/286 (0.0%)	0/2608 (0.0%)	NA
Pneumonia	0/3437 (0.0%)	0/360 (0.0%)	0/3077 (0.0%)	NA	0/543 (0.0%)	0/74 (0.0%)	0/469 (0.0%)	NA	0/2894 (0.0%)	0/286 (0.0%)	0/2608 (0.0%)	NA
Other	54/3436 (1.6%)	10/360 (2.8%)	44/3076 (1.4%)	0.052(NS)	7/543 (1.3%)	2/74 (2.7%)	5/469 (1.1%)	0.245(NS)†	47/2893 (1.6%)	8/286 (2.8%)	39/2607 (1.5%)	0.131(NS)†

**Table 6:** Adverse events at 1 year follow-up

	Cryo or RF energy			*p*	Cryo energy			*p*	RF energy			*p*
	Total	Adenosine	No adenosine		Total	Adenosine	No adenosine		RF	Adenosine	No adenosine	
Permanent pacemaker implantation	23/131 (17.6%)	1/15 (6.7%)	22/116 (19.0%)	0.468(NS)	0/24 (0.0%)	0/5 (0.0%)	0/19 (0.0%)	NA	23/107 (21.5%)	1/10 (10.0%)	22/97 (22.7%)	0.686(NS)
*Cardiovascular*	31/3057 (1.0%)	3/314 (1.0%)	28/2743 (1.0%)	1.000(NS)	3/494 (0.6%)	0/71 (0.0%)	3/423 (0.7%)	1.000(NS)	28/2563 (1.1%)	3/243 (1.2%)	25/2320 (1.1%)	0.744(NS)
Cardiac Arrest	0/3057 (0.0%)	0/314 (0.0%)	0/2743 (0.0%)	NA	0/494 (0.0%)	0/71 (0.0%)	0/423 (0.0%)	NA	0/2563 (0.0%)	0/243 (0.0%)	0/2320 (0.0%)	NA
Air Embolism	1/3057 (0.0%)	0/314 (0.0%)	1/2743 (0.0%)	1.000(NS)	0/494 (0.0%)	0/71 (0.0%)	0/423 (0.0%)	NA	1/2563 (0.0%)	0/243 (0.0%)	1/2320 (0.0%)	1.000(NS)
Endocarditis	1/3057 (0.0%)	0/314 (0.0%)	1/2743 (0.0%)	1.000(NS)	0/494 (0.0%)	0/71 (0.0%)	0/423 (0.0%)	NA	1/2563 (0.0%)	0/243 (0.0%)	1/2320 (0.0%)	1.000(NS)
Myocardial Infarction	2/3057 (0.1%)	0/314 (0.0%)	2/2743 (0.1%)	1.000(NS)	0/494 (0.0%)	0/71 (0.0%)	0/423 (0.0%)	NA	2/2563 (0.1%)	0/243 (0.0%)	2/2320 (0.1%)	1.000(NS)
Cardiac perforation	6/3057 (0.2%)	0/314 (0.0%)	6/2743 (0.2%)	1.000(NS)	1/494 (0.2%)	0/71 (0.0%)	1/423 (0.2%)	1.000(NS)	5/2563 (0.2%)	0/243 (0.0%)	5/2320 (0.2%)	1.000(NS)
Bradycardia Requiring Pacemaker	5/3057 (0.2%)	0/314 (0.0%)	5/2743 (0.2%)	1.000(NS)	0/494 (0.0%)	0/71 (0.0%)	0/423 (0.0%)	NA	5/2563 (0.2%)	0/243 (0.0%)	5/2320 (0.2%)	1.000(NS)
Cardiac Thromboembolic Event	0/3057 (0.0%)	0/314 (0.0%)	0/2743 (0.0%)	NA	0/494 (0.0%)	0/71 (0.0%)	0/423 (0.0%)	NA	0/2563 (0.0%)	0/243 (0.0%)	0/2320 (0.0%)	NA
Heart Valve Damage	1/3057 (0.0%)	0/314 (0.0%)	1/2743 (0.0%)	1.000(NS)	0/494 (0.0%)	0/71 (0.0%)	0/423 (0.0%)	NA	1/2563 (0.0%)	0/243 (0.0%)	1/2320 (0.0%)	1.000(NS)
Pericarditis	4/3057 (0.1%)	1/314 (0.3%)	3/2743 (0.1%)	0.352(NS)	0/494 (0.0%)	0/71 (0.0%)	0/423 (0.0%)	NA	4/2563 (0.2%)	1/243 (0.4%)	3/2320 (0.1%)	0.329(NS)
Other	14/3057 (0.5%)	2/314 (0.6%)	12/2743 (0.4%)	0.648(NS)	1/494 (0.2%)	0/71 (0.0%)	1/423 (0.2%)	1.000(NS)	13/2563 (0.5%)	2/243 (0.8%)	11/2320 (0.5%)	0.353(NS)
*General*	3/3057 (0.1%)	1/314 (0.3%)	2/2743 (0.1%)	0.278(NS)	1/494 (0.2%)	1/71 (1.4%)	0/423 (0.0%)	0.144(NS)	2/2563 (0.1%)	0/243 (0.0%)	2/2320 (0.1%)	1.000(NS)
Allergic Reaction	2/3057 (0.1%)	1/314 (0.3%)	1/2743 (0.0%)	0.195(NS)	1/494 (0.2%)	1/71 (1.4%)	0/423 (0.0%)	0.144(NS)	1/2563 (0.0%)	0/243 (0.0%)	1/2320 (0.0%)	1.000(NS)
Sepsis	0/3057 (0.0%)	0/314 (0.0%)	0/2743 (0.0%)	NA	0/494 (0.0%)	0/71 (0.0%)	0/423 (0.0%)	NA	0/2563 (0.0%)	0/243 (0.0%)	0/2320 (0.0%)	NA
*Gastrointestinal*	4/3057 (0.1%)	1/314 (0.3%)	3/2743 (0.1%)	0.352(NS)	2/494 (0.4%)	1/71 (1.4%)	1/423 (0.2%)	0.267(NS)	2/2563 (0.1%)	0/243 (0.0%)	2/2320 (0.1%)	1.000(NS)
Esophageal Fistula or perforation	0/3057 (0.0%)	0/314 (0.0%)	0/2743 (0.0%)	NA	0/494 (0.0%)	0/71 (0.0%)	0/423 (0.0%)	NA	0/2563 (0.0%)	0/243 (0.0%)	0/2320 (0.0%)	NA
Esophageal ulceration	0/3057 (0.0%)	0/314 (0.0%)	0/2743 (0.0%)	NA	0/494 (0.0%)	0/71 (0.0%)	0/423 (0.0%)	NA	0/2563 (0.0%)	0/243 (0.0%)	0/2320 (0.0%)	NA
Neurological	8/3057 (0.3%)	3/314 (1.0%)	5/2743 (0.2%)	0.041(S)	3/494 (0.6%)	2/71 (2.8%)	1/423 (0.2%)	0.055(NS)	5/2563 (0.2%)	1/243 (0.4%)	4/2320 (0.2%)	0.393(NS)
Stroke	1/3057 (0.0%)	0/314 (0.0%)	1/2743 (0.0%)	1.000(NS)	0/494 (0.0%)	0/71 (0.0%)	0/423 (0.0%)	NA	1/2563 (0.0%)	0/243 (0.0%)	1/2320 (0.0%)	1.000(NS)
TIA	5/3057 (0.2%)	1/314 (0.3%)	4/2743 (0.1%)	0.419(NS)	1/494 (0.2%)	0/71 (0.0%)	1/423 (0.2%)	1.000(NS)	4/2563 (0.2%)	1/243 (0.4%)	3/2320 (0.1%)	0.329(NS)
Phrenic Nerve Damage	2/2973 (0.1%)	2/304 (0.7%)	0/2669 (0.0%)	0.010(S)	2/480 (0.4%)	2/68 (2.9%)	0/412 (0.0%)	0.020(S)	0/2493 (0.0%)	0/236 (0.0%)	0/2257 (0.0%)	NA
Vagal nerve injury	0/3057 (0.0%)	0/314 (0.0%)	0/2743 (0.0%)	NA	0/494 (0.0%)	0/71 (0.0%)	0/423 (0.0%)	NA	0/2563 (0.0%)	0/243 (0.0%)	0/2320 (0.0%)	NA
Peripheral nerve injury	0/3057 (0.0%)	0/314 (0.0%)	0/2743 (0.0%)	NA	0/494 (0.0%)	0/71 (0.0%)	0/423 (0.0%)	NA	0/2563 (0.0%)	0/243 (0.0%)	0/2320 (0.0%)	NA
*Peripheral-vascular*	21/3057 (0.7%)	1/314 (0.3%)	20/2743 (0.7%)	0.717(NS)	5/494 (1.0%)	0/71 (0.0%)	5/423 (1.2%)	1.000(NS)	16/2563 (0.6%)	1/243 (0.4%)	15/2320 (0.6%)	1.000(NS)
AV fistula	8/2973 (0.3%)	0/304 (0.0%)	8/2669 (0.3%)	1.000(NS)	3/480 (0.6%)	0/68 (0.0%)	3/412 (0.7%)	1.000(NS)	5/2493 (0.2%)	0/236 (0.0%)	5/2257 (0.2%)	1.000(NS)
DVT	2/3057 (0.1%)	0/314 (0.0%)	2/2743 (0.1%)	1.000(NS)	0/494 (0.0%)	0/71 (0.0%)	0/423 (0.0%)	NA	2/2563 (0.1%)	0/243 (0.0%)	2/2320 (0.1%)	1.000(NS)
Peripheral thromboembolic event	1/3057 (0.0%)	0/314 (0.0%)	1/2743 (0.0%)	1.000(NS)	0/494 (0.0%)	0/71 (0.0%)	0/423 (0.0%)	NA	1/2563 (0.0%)	0/243 (0.0%)	1/2320 (0.0%)	1.000(NS)
Pseudoaneurysm	9/2973 (0.3%)	1/304 (0.3%)	8/2669 (0.3%)	1.000(NS)	3/480 (0.6%)	0/68 (0.0%)	3/412 (0.7%)	1.000(NS)	6/2493 (0.2%)	1/236 (0.4%)	5/2257 (0.2%)	0.450(NS)
Hematoma or bleeding requiring evacuation or transfusion	3/3057 (0.1%)	0/314 (0.0%)	3/2743 (0.1%)	1.000(NS)	0/494 (0.0%)	0/71 (0.0%)	0/423 (0.0%)	NA	3/2563 (0.1%)	0/243 (0.0%)	3/2320 (0.1%)	1.000(NS)
Bleeding requiring transfusion	0/3057 (0.0%)	0/314 (0.0%)	0/2743 (0.0%)	NA	0/494 (0.0%)	0/71 (0.0%)	0/423 (0.0%)	NA	0/2563 (0.0%)	0/243 (0.0%)	0/2320 (0.0%)	NA
*Pulmonary*	10/3057 (0.3%)	1/314 (0.3%)	9/2743 (0.3%)	1.000(NS)	2/494 (0.4%)	1/71 (1.4%)	1/423 (0.2%)	0.267(NS)	8/2563 (0.3%)	0/243 (0.0%)	8/2320 (0.3%)	1.000(NS)
Hemotorax	0/3057 (0.0%)	0/314 (0.0%)	0/2743 (0.0%)	NA	0/494 (0.0%)	0/71 (0.0%)	0/423 (0.0%)	NA	0/2563 (0.0%)	0/243 (0.0%)	0/2320 (0.0%)	NA
Pneumotorax	3/3057 (0.1%)	0/314 (0.0%)	3/2743 (0.1%)	1.000(NS)	0/494 (0.0%)	0/71 (0.0%)	0/423 (0.0%)	NA	3/2563 (0.1%)	0/243 (0.0%)	3/2320 (0.1%)	1.000(NS)
Pulmonary vein damage/dissection	0/3057 (0.0%)	0/314 (0.0%)	0/2743 (0.0%)	NA	0/494 (0.0%)	0/71 (0.0%)	0/423 (0.0%)	NA	0/2563 (0.0%)	0/243 (0.0%)	0/2320 (0.0%)	NA
Pulmonary vein stenosis	4/3057 (0.1%)	0/314 (0.0%)	4/2743 (0.1%)	1.000(NS)	0/494 (0.0%)	0/71 (0.0%)	0/423 (0.0%)	NA	4/2563 (0.2%)	0/243 (0.0%)	4/2320 (0.2%)	1.000(NS)
Pleural Effusion	0/3057 (0.0%)	0/314 (0.0%)	0/2743 (0.0%)	NA	0/494 (0.0%)	0/71 (0.0%)	0/423 (0.0%)	NA	0/2563 (0.0%)	0/243 (0.0%)	0/2320 (0.0%)	NA
Pulmonary embolism	0/3057 (0.0%)	0/314 (0.0%)	0/2743 (0.0%)	NA	0/494 (0.0%)	0/71 (0.0%)	0/423 (0.0%)	NA	0/2563 (0.0%)	0/243 (0.0%)	0/2320 (0.0%)	NA
Pulmonary vein thrombosis	0/3057 (0.0%)	0/314 (0.0%)	0/2743 (0.0%)	NA	0/494 (0.0%)	0/71 (0.0%)	0/423 (0.0%)	NA	0/2563 (0.0%)	0/243 (0.0%)	0/2320 (0.0%)	NA
Pneumonia	3/3057 (0.1%)	1/314 (0.3%)	2/2743 (0.1%)	0.278(NS)	2/494 (0.4%)	1/71 (1.4%)	1/423 (0.2%)	0.267(NS)	1/2563 (0.0%)	0/243 (0.0%)	1/2320 (0.0%)	1.000(NS)
*Other*	48/3057 (1.6%)	6/314 (1.9%)	42/2743 (1.5%)	0.628(NS)	9/494 (1.8%)	1/71 (1.4%)	8/423 (1.9%)	1.000(NS)	39/2563 (1.5%)	5/243 (2.1%)	34/2320 (1.5%)	0.411(NS)

*AV* arteriovenous, *DVT* deep vein thrombosis, *NA* not applicable, *NS* non significant, *RF* radio frequency, *S* significant, *TIA* transient ischemic attack
